# Comparison of effects of diet versus exercise weight loss regimens on LDL and HDL particle size in obese adults

**DOI:** 10.1186/1476-511X-10-119

**Published:** 2011-07-18

**Authors:** Krista A Varady, Surabhi Bhutani, Monica C Klempel, Cynthia M Kroeger

**Affiliations:** 1 Department of Kinesiology and Nutrition, University of Illinois at Chicago, Chicago, IL, USA

**Keywords:** Calorie restriction, alternate day fasting, endurance exercise, weight loss, LDL particle size, HDL particle size, cholesterol, obese adults

## Abstract

**Background:**

Obesity is associated with an atherogenic lipid profile characterized by a predominance of small LDL and HDL particles. Weight loss, by dietary restriction or exercise, increases LDL particle size. Whether these interventions can augment HDL size *in conjunction *with LDL size remains unknown.

**Objective:**

This study compared the effects of alternate day fasting (ADF), calorie restriction (CR), and endurance exercise on LDL and HDL particle size in overweight and obese subjects.

**Methods:**

In a 12-week parallel-arm trial, adult subjects (n = 60) were randomized to 1 of 4 groups: 1) ADF (75% energy restriction for 24-h alternated with ad libitum feeding for 24-h), 2) CR (25% energy restriction every day), 3) exercise (moderate intensity training 3 x/week), or 4) control.

**Results:**

Body weight was reduced (*P *< 0.001) by ADF, CR, and exercise (5.2 ± 1.1%, 5.0 ± 1.4%, 5.1 ± 0.9%, respectively). Plasma LDL cholesterol decreased (*P *< 0.05) with ADF (10 ± 4%) and CR (8 ± 4%), whereas HDL cholesterol increased (*P *< 0.05) with exercise (16 ± 5%). Integrated LDL particle size was augmented (*P *= 0.01) by ADF and CR. The proportion of small LDL particles decreased (*P *= 0.04) with ADF only, and the proportion of large HDL particles increased (*P *= 0.03) with exercise only.

**Conclusion:**

These results indicate that dietary restriction increases LDL particle size, while endurance training augments HDL particle size, with minimal weight loss. None of these interventions concomitantly increased both LDL and HDL particle size, however.

## Introduction

Epidemiological evidence suggests that an increased proportion of small, dense LDL and HDL particles is strongly associated with the risk of coronary heart disease (CHD) [1-3]. Potential mechanisms that link small LDL particles to increased risk of vascular events include: augmented oxidizability [4] and increased permeability through the endothelial barrier [5]. As for HDL particle size, the mechanisms that link this parameter to increased CHD risk have yet to be firmly established, but may involve the altered activity of lipases involved with the maturation and transformation of lipoproteins [6].

Overweight and obesity are associated with an atherogenic lipoprotein phenotype that is characterized by the predominance of small LDL and HDL particles [7]. Weight loss, by means of dietary restriction or exercise, has been shown to decrease the proportion of small LDL particles [8]. Two forms of dietary restriction currently implemented include calorie restriction (CR) and alternate day fasting (ADF) [9]. CR regimens involve reducing energy intake by 15-40% of needs every day, whereas ADF regimens involve a day of partial energy restriction alternated with a day of ad libitum feeding. Both diets effectively reduce the proportion of small LDL particles once 5% weight loss is achieved [10,11]. Moderate intensity endurance training is another lifestyle strategy that has been shown to decrease the concentration of small LDL particles with 5% weight loss [12-14]. As for the effect of these interventions on HDL particle size, data are very limited. For instance, to our knowledge, no studies to date have examined the impact of either ADF or CR on HDL size. In terms of the effect of exercise on this CHD risk parameter, two recent reports demonstrate increases in HDL size after short intervention periods [15,16]. A key question that remains unresolved is whether these lifestyle therapies (i.e. diet versus exercise) are able to beneficially modulate HDL particle size *in conjunction *with LDL particle size. Given that small LDL and HDL particles are key risk factors for CHD [1-3], a lifestyle regimen that could beneficially modulate *both *of these parameters may be considered highly cardio-protective. Accordingly, the objective of the present study was to compare the effects of ADF, CR, and endurance exercise on changes in LDL and HDL particle size in overweight and obese subjects when a similar degree of weight loss is achieved.

## Materials and methods

### Study design and subject selection

A 12-week, randomized, controlled, parallel-arm trial was implemented as a means of testing the study objectives. Overweight and obese subjects (n = 60) were randomized into 1 of 4 groups: (1) ADF, n = 15, (2) CR, n = 15, (3) exercise, n = 15, or (4) control, n = 15. Key inclusion criteria were as follows: body mass index (BMI) between 25 and 39.9 kg/m^2^; age 35 to 65 y; non-diabetic; no history of cardiovascular disease; non-smoker; weight stable (<6 kg weight loss or gain for 3 months prior to the study); sedentary or lightly active (<3 h/week of light intensity exercise for 3 months prior to the study); and not taking weight loss, lipid, or glucose lowering medications. The experimental protocol was approved by the Office for the Protection of Research Subjects at the University of Illinois, Chicago. All volunteers gave their written informed consent to participate in the trial.

### Diet protocol

Only the ADF and CR groups participated in the diet intervention. Baseline energy needs were assessed using the Mifflin equation [17]. ADF subjects were restricted by 75% of their baseline needs on the fast day, and ate ad libitum on each alternating feed day. The feed/fast days began at midnight each day, and all fast day meals were consumed between 12.00 pm and 2.00 pm. ADF subjects were provided with calorie-restricted meals on each fast day, and ate ad libitum at home on the feed day. CR subjects were restricted by 25% of their baseline energy needs each day, and were provided with all of their calorie-restricted meals throughout the trial. Study diets were provided as a 3-day rotating menu, and were formulated based on the American Heart Association guidelines (30% kcal from fat, 15% kcal from protein, 55% kcal from carbohydrate). Subjects in the control and exercise groups were permitted to eat ad libitum every day, and were not provided with meals from the research center. The ADF and CR diets were designed to produce ~5% weight loss after 12 weeks of treatment.

### Exercise protocol

Only the exercise group participated in the training intervention. These subjects participated in a moderate intensity exercise program 3 times per week under supervised conditions, for 12 weeks. Exercise was performed using stationary bikes and elliptical machines. Training intensity was estimated for each individual using an age-predicted heart rate maximum (HRmax) equation [209 − (0.7 × age)] [18] and a Polar Heart Rate Monitor (Polar USA, Inc., NY). At the beginning of the study, each exercise session ran for 45 min and correspond to 60% of the subject's HRmax. Training duration and intensity was then increased incrementally at week 4, 7 and 10 by 5 min and 5% HRmax. As such by week 10, each subject was exercising for 60 min at an intensity of 75% HRmax. Subjects in the ADF, CR, and control groups were asked to maintain their regular level of activity throughout the course of the trial.

### Biochemical measures

Twelve-hour fasting blood samples were collected at baseline (week 1) and post-treatment (week 12). Plasma total cholesterol, direct LDL-cholesterol, HDL-cholesterol, and triglyceride concentrations were measured in duplicate using enzymatic kits (Biovision Inc., Moutainview, CA, USA). LDL and HDL particle size were measured by linear polyacrylamide gel electrophoresis [19] (Quantimetrix Lipoprint System, Redondo Beach, CA). High-resolution 3% polyacrylamide gel tubes were used for electrophoresis. Briefly, 25 µL of sample was mixed with 200 µL of liquid loading gel containing Sudan black, and added to the gel tubes. After photopolymerization at room temperature for 30 min, samples were electrophoresed for 1 h (3 mA/gel tube). Lipoware computer software (Quantimetrix, Redondo Beach, CA) was then used to divide LDL into small (<255 Å), medium (255-260 Å), and large (>260 Å) particles, and HDL into small (<73 Å), medium (73-88 Å), and large (>88 Å) particles [19].

### Statistics

Results are presented as mean ± SEM. Differences between groups at baseline and post-treatment were tested by one-way ANOVA followed by post hoc Tukey's tests. Changes from baseline to week 12 within an intervention group were tested by Student's paired t-test. Baseline values of the subjects who did not complete the trial were included in the analysis. *P *values < 0.05 were considered significant. Data were analyzed using SPSS software (version 18.0 for Mac OS X; SPSS Inc., Chicago, IL).

## Results

### Baseline characteristics and weight loss

Sixty subjects commenced the study, with 49 completing the 12-week trial. The remaining subjects in each intervention group were as follows: ADF (n = 13), CR (n = 12), exercise (n = 12), and control (n = 12). There were no significant differences at baseline between the ADF, CR, exercise, and control groups, respectively, with regards to age (47 ± 2, 47 ± 3, 46 ± 3, 46 ± 3 y), sex (male/female: 3/10, 2/10, 2/10, 2/10), BMI (32 ± 2, 32 ± 2, 33 ± 1, 32 ± 2 kg/m^2^), LDL cholesterol concentrations (141 ± 9, 137 ± 9, 122 ± 9, 136 ± 10 mg/dL), or HDL cholesterol concentrations (51 ± 3, 60 ± 6, 51 ± 4, 57 ± 3 mg/dL). After 12 weeks, body weight decreased (*P *< 0.001) in the ADF, CR, and exercise groups (5.2 ± 1.1%, 5.0 ± 1.4%, 5.1 ± 0.9%, respectively), but remained stable (-0.2 ± 0.4%) in the control group.

### Plasma lipid concentrations

**Figure 1:**
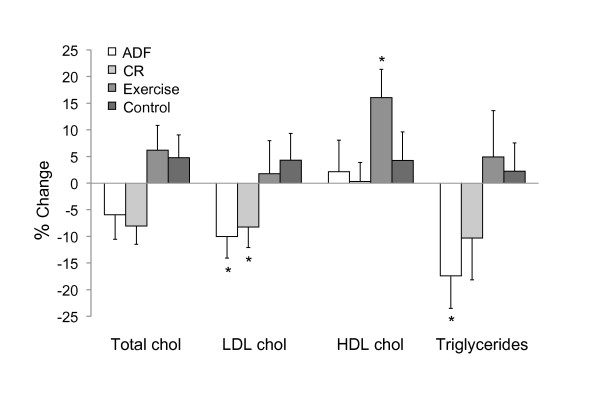
**Change in plasma lipid concentrations after 12 weeks of treatment**. Values reported as mean ± SEM. Alternate day fasting (ADF), n = 13; Calorie restriction (CR), n = 12; Exercise, n = 12; Control, n = 12. *Significantly different between groups, *P <*0.05 (One-way ANOVA).

Total cholesterol concentrations did not change over the course of the trial (Figure 1). LDL cholesterol concentrations decreased (*P *< 0.05) with ADF (10 ± 4%) and CR (8 ± 4%) only, whereas HDL cholesterol concentrations increased (*P *< 0.05) with exercise (16 ± 5%) only. Triglycerides were reduced (*P *< 0.05) in the ADF group (17 ± 5%), but were not affected by any other intervention.

### LDL and HDL particle size

Integrated LDL particle size increased (*P *= 0.01) in response to ADF and CR only (Table 1). The proportion of large LDL particles increased (*P *= 0.04) while the proportion of small particles decreased (*P *= 0.04) in the ADF group only. There was no effect of CR or exercise on LDL particle distribution. Only the exercise group experienced increases (*P *= 0.04) in the proportion of large HDL particles, and decreases (*P *= 0.03) in the proportion of small HDL particles.

**Table 1:** LDL and HDL particle size and distribution at baseline and post-treatment

	Group	Week 1 ^a^		Week 12		P-value within group ^b^	P-value between groups ^c^
		**Mean**	**SEM**	**Mean**	**SEM**		

LDL integrated particle size (Å)	ADF	261	± 1	265	± 2	0.01	0.20
	CR	260	± 2	264	± 2	0.01	
	Exercise	260	± 2	261	± 1	0.70	
	Control	260	± 2	260	± 2	0.49	
							
% Large LDL particles	ADF	40	± 4	48	± 5	0.04	0.43
	CR	47	± 5	52	± 5	0.07	
	Exercise	49	± 4	49	± 4	0.49	
	Control	42	± 4	41	± 4	0.53	
							
% Medium LDL particles	ADF	35	± 2	34	± 2	0.75	0.49
	CR	36	± 3	34	± 3	0.57	
	Exercise	35	± 2	34	± 2	0.48	
	Control	39	± 2	39	± 2	0.87	
							
% Small LDL particles	ADF	25	± 3	18	± 3	0.04	0.75
	CR	18	± 3	14	± 4	0.29	
	Exercise	17	± 3	17	± 3	0.89	
	Control	19	± 4	20	± 4	0.50	
							
% Large HDL particles	ADF	34	± 3	34	± 3	0.93	0.98
	CR	34	± 4	36	± 5	0.67	
	Exercise	28	± 3	34	± 3	0.04	
	Control	34	± 4	34	± 5	0.98	
							
% Medium HDL particles	ADF	49	± 3	46	± 3	0.39	0.31
	CR	43	± 4	44	± 4	0.85	
	Exercise	52	± 3	52	± 3	0.80	
	Control	45	± 3	44	± 4	0.64	
							
% Small HDL particles	ADF	18	± 3	20	± 3	0.61	0.53
	CR	22	± 4	20	± 6	0.57	
	Exercise	21	± 4	14	± 3	0.03	
	Control	21	± 3	22	± 3	0.60	

Values reported as mean ± SEM.

Alternate day fasting (ADF), n = 13; Calorie restriction (CR), n = 12; Exercise, n = 12; Control, n = 12.

^a ^Baseline values were not significantly different between intervention groups: One-way ANOVA.

^b ^P-value within group: Student's paired t-test comparing week 1 to week 12 values.

^c ^P-value between groups: One-way ANOVA between groups for week 12 values.

## Discussion

This study is the first to show that diet and exercise interventions that achieve similar degrees of weight loss have differential effects on LDL versus HDL particle size. More specifically, we show here that ADF and CR regimens that achieve 5% weight loss increase LDL size, but have no effect on HDL size. In contrast, exercise training that results in 5% weight loss increases the proportion of large HDL particles, but has no impact on LDL size. Thus, none of these interventions are able to beneficially modulate *both *LDL and HDL particle size with only a minimal amount of weight loss.

These findings for the effects of ADF and CR on LDL subclasses are in line with what has been reported previously [10,11]. For instance, in a recent trial by Morgan et al. [10] it was shown that 6% weight loss by CR increased LDL particle size in obese adults after 8 weeks. Similarly, in a trial by Varady et al. [11], 5% weight loss by ADF decreased the proportion of small LDL particles in obese volunteers after 8 weeks of treatment. The present study adds to this body of evidence by demonstrating that ADF may be *more effective *than CR at modulating LDL particle size. For instance, we show here that ADF was able to increase the proportion of large LDL particles while decreasing the proportion of small LDL particles. Conversely, these effects on LDL distribution were not demonstrated by CR. Since a decrease in the proportion of small LDL particles is a stronger predictor of CHD risk than LDL integrated size [20], these results suggest that ADF may confer added protection against CHD when compared to CR. The mechanisms whereby ADF and CR confer different effects on LDL particle size are unknown at present, but will be an interesting area of future investigation in this field. These two diets were similar, however, in that neither had any impact on HDL particle size distribution. Although the reason for this lack of effect is not clear, it is possible that a greater degree of weight loss (i.e. 10-15%) may be required to modulate HDL particle size. Since this is first study to test the effects of ADF and CR on HDL particle size, there are no studies to which to compare the present findings.

The impact of 12 weeks of endurance exercise on HDL and LDL size was also investigated. We show here that exercise beneficially modulated HDL particle distribution by decreasing the proportion of small HDL particles and increasing the proportion of large HDL particles. These results are complementary to other recent reports. For example, Brown et al. [15] demonstrated significant decreases in the proportion of small HDL particles after 12 weeks of endurance training in obese women with only 2% weight loss. Likewise, Obisesan et al. [16] observed significant increases the proportion of large HDL particles with 24 weeks of endurance training in overweight adults with 6% weight loss. As for LDL particle size, no beneficial exercise-induced effects were noted in the present study, which is in contrast to what has been demonstrated previously [12-14]. A couple of factors may account for the lack of effect of exercise on LDL subfractions. Firstly, our intervention period may have been too short to observe an effect since previous work only demonstrates decreases in LDL particle size with >24 weeks of training [12-14]. Secondly, the intensity of our exercise program may have not been sufficient (i.e. 60-75% HRmax), since decreases in LDL size are generally only shown with training intensities >75% HRmax [12-14]. It will therefore be important for upcoming trials testing similar objectives to take into account these minimal requirements for trial length and training intensity.

In summary, these results indicate that dietary restriction is able to markedly increase LDL particle size, while endurance training is able to augment HDL particle size, with minimal weight loss (5% from baseline). None of these interventions were able to modulate *both *LDL and HDL size, however. Nevertheless, since these lifestyle strategies appear to have complementary effects, it will be of interest for future studies to test whether concomitant increases in HDL and LDL particle size can be attained when ADF or CR is combined with exercise.

## Competing interests

The authors declare that they have no competing interests.

## Authors' contributions

KAV designed the study and prepared the manuscript. SB, MCK, and CMK coordinated the clinical trial and performed the laboratory analyses. All authors read and approved the final manuscript.
